# Open Intraperitoneal Onlay Mesh (IPOM) Technique for Incisional Hernia Repair

**DOI:** 10.3389/fsurg.2018.00066

**Published:** 2018-10-23

**Authors:** Ferdinand Köckerling, Bernhard Lammers

**Affiliations:** ^1^Department of Surgery and Center for Minimally Invasive Surgery, Academic Teaching Hospital of Charité Medical School, Vivantes Hospital, Berlin, Germany; ^2^Department of Surgery I – Section Coloproctologie and Hernia Surgery, Lukas Hospital, Neuss, Germany

**Keywords:** incisional hernia, open intraperitoneal onlay, IPOM, surgical site infection, seroma, wound complication

## Abstract

In an Expert Consensus Guided by Systematic Review the panel agreed that for open elective incisional hernia repair sublay mesh location is preferred, but open intraperitoneal onlay mesh (IPOM) may be useful in certain settings. Accordingly, the available literature on the open IPOM technique was searched and evaluated.

**Material and Methods:** A systematic search of the available literature was performed in July 2018 using Medline, PubMed, and the Cochrane Library. Forty-five publications were identified as relevant for the key question.

**Results:** Compared to laparoscopic IPOM, the open IPOM technique was associated with significantly higher postoperative complication rates and recurrence rates. For the open IPOM with a bridging situation the postoperative complication rate ranges between 3.3 and 72.0% with a mean value of 20.4% demonstrating high variance, as did the recurrence rate of between 0 and 61.0% with a mean value of 12.6%. Only on evaluation of the upward-deviating maximum values and registry data is a trend toward better outcomes for the sublay technique demonstrated. Through the use of a wide mesh overlap, avoidance of dissection in the abdominal wall and defect closure it appears possible to achieve better outcomes for the open IPOM technique.

**Conclusion:** Compared to the laparoscopic technique, open IPOM is associated with significantly poorer outcomes. For the sublay technique the outcomes are quite similar and only tendentially worse. Further studies using an optimized open IPOM technique are urgently needed.

## Introduction

Two recently published systematic reviews and meta-analyses and a registry study once again impressively demonstrated that for incisional hernias mesh techniques compared with suture techniques resulted in significantly lower recurrence rates (1–3). However, which mesh technique assures the best outcomes for the respective patient is still under debate.

While meta-analyses have identified advantages for the laparoscopic compared with the open technique for repair of incisional hernia (4–7), in the guidelines the laparoscopic intraperitoneal onlay mesh (IPOM) technique is recommended only for a defect size of up to 10 cm (8–12).

In an Expert Consensus Guided by Systematic Review the panel agreed that for open elective incisional hernia repair sublay mesh location is preferred, but open intraperitoneal onlay mesh (IPOM) may be useful in certain settings (13). In systematic reviews the open IPOM technique is discussed, in particular, in the context of large incisional hernias (14, 15). Based on the expert consensus, this paper now explores and evaluates the available literature on open intraperitoneal onlay mesh (IPOM) in accordance with the Parker (16) nomenclature.

## Materials and methods

**Figure 1:**
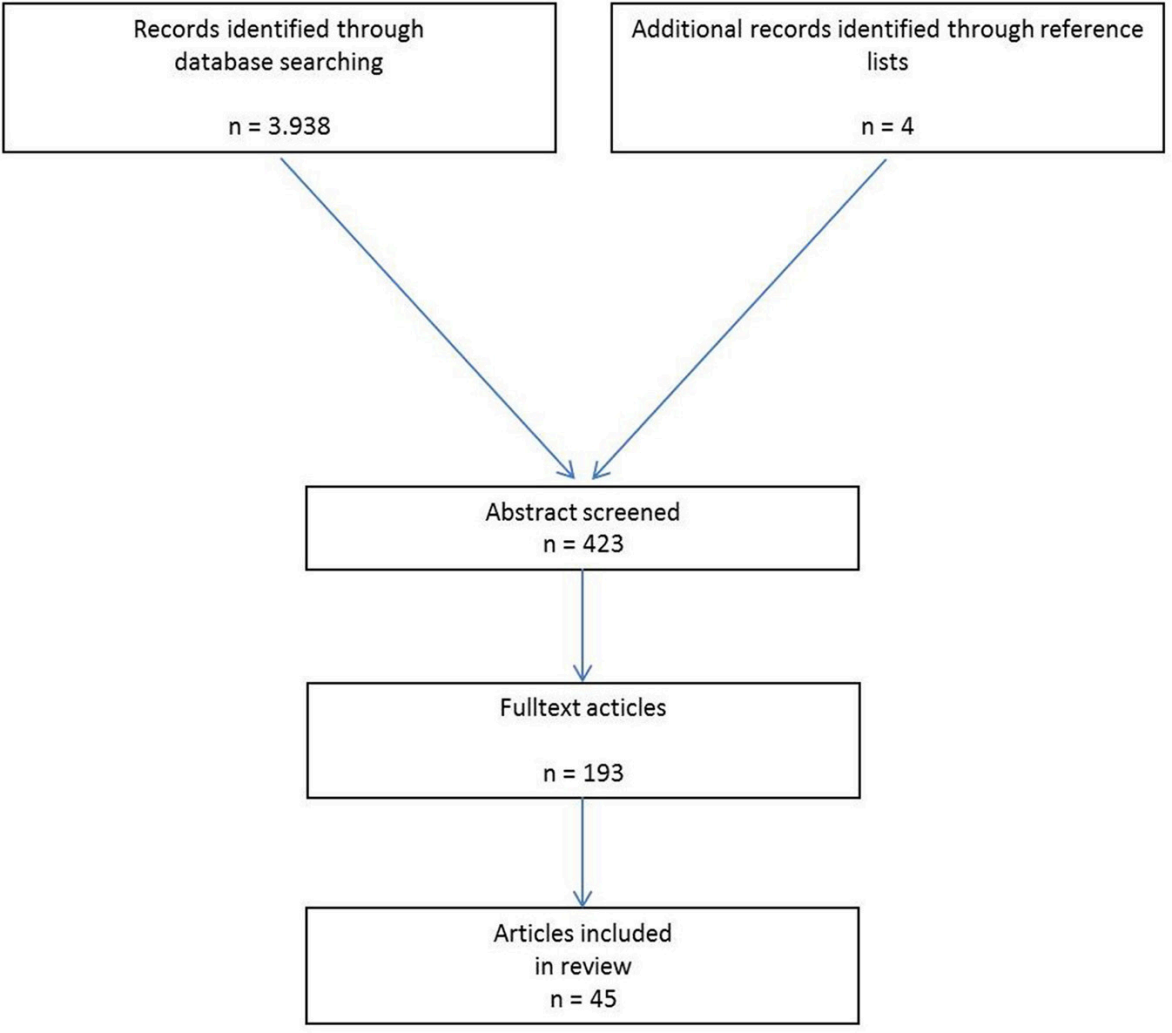
Flowchart of article inclusion.

A systematic search of the available literature was performed in July 2018 using Medline, PubMed, and the Cochrane Library, as well as a search of relevant journals and reference lists. The following search terms were used: “Incisional hernia,” “Intraperitoneal mesh,” “Open intraperitoneal onlay mesh,” “Open IPOM,” and “IPOM.” The abstracts of 423 publications were checked. For the present analysis 45 publications were identified as relevant for the key question (Figure 1).

## Results

### Laparoscopic vs. open intraperitoneal onlay mesh (IPOM) with bridging for incisional hernia repair

In a prospective (17) and a retrospective study (18) the outcomes of the laparoscopic and open IPOM techniques for incisional hernia were compared (Table 1). For the prospective study comparison of 127 laparoscopic and 233 open IPOM incisional hernias repairs showed a significantly lower postoperative complication rate (Clavien-Dindo ≥ II) of 7% for the laparoscopic and of 15% for the open IPOM technique (*p* < 0.01) (17).

**Table 1:** Laparoscopic vs. open intraperitoneal onlay mesh for incisional hernia repair.

**Authors**	**Study design**	**Patients**	**Hernia type**	**Inclusion/Exclusion**	**Mesh type**	**Postoperative complications**	**Hospital stay**	**Recurrence**
(17)	Prospective comparative study	Laparoscopic IPOM *n* = 127 Open IPOM *n* = 233	Incisional	Patients with additional procedures were excluded	PTFE, Polypropylene, Combination	Open IPOM Clavien-Dindo ≥ II: 15% Laparoscopic IPOM Clavien-Dindo ≥ II: 7% *p* < 0.01	Mean: Open IPOM 1.4 ± 2.0 days vs. Laparoscopic IPOM 0.9 ± 1.4 days *p* = 0.01	Open IPOM: 9% in mean follow-up of 36 months vs. Laparoscopic IPOM: 13% in mean follow-up of 30 months *p* = 0.36
(18)	Retrospective comparative study	Laparoscopic IPOM *n* = 120 Open IPOM *n* = 64	Incisional	Primary incision median (70%), transverse (25%), other (5%)	Parietene composite 68%, Parietex composite 15%, DynaMesh 9%, other 8%	Open IPOM 23% vs. Laparoscopic IPOM 10% *p* = 0.046	Median: Open IPOM 8 (5–12) days vs. Laparoscopic IPOM 6 (4–7) days *p* = 0.002	Open IPOM 19% vs. Laparoscopic IPOM 20% after a mean follow-up of 5.5 years *p* = ns

Likewise, the hospital stay for the laparoscopic IPOM was significantly shorter (0.9 ± 1.4 days vs. 1.4 ± 2.0 days; *p* = 0.01) (17). No significant difference was seen in the recurrence rate (laparoscopic 13% vs. open 9%; *p* = 0.36) (17).

Similarly, the retrospective comparative study of 120 laparoscopic and 64 open IPOM operations showed comparable outcomes (18), with a significantly lower postoperative complication rate in favor of the laparoscopic technique (10 vs. 23%; *p* = 0.046) (18). Similarly, in this study the hospital stay after laparoscopic IPOM was significantly shorter [median 6 (4–7) days vs. 8 (5–12) days; *p* = 0.002] (18). Likewise, in this study no significant difference was found in the recurrence rate after laparoscopic vs. open IPOM for incisional hernia repairs (laparoscopic 20% vs. open 19%; *p* = ns) (18).

Hence, the laparoscopic compared with the open IPOM technique had a significantly lower postoperative complication rate and hospital stay and with a somewhat similar recurrence rate.

Accordingly, for defects up for 10 cm preference should in general be given to laparoscopic over open IPOM repair.

### Open intraperitoneal onlay mesh with bridging in incisional hernia repair

Since in general open IPOM is indicated for incisional hernia repair of large defects when a sublay technique is no longer feasible, this mainly involves a bridging situation. Accordingly, most of the studies published report on an open intraperitoneal onlay mesh technique with bridging (19–28) (Table 2). The outcomes demonstrated high variance (Table 2). The postoperative complication rates were in the range of 3.3–72% and the recurrence rates 0–61%. The mean value calculated for the open intraperitoneal onlay mesh technique in the studies featured in Table 2 was 20.4% (range: 3.3–72%) for the postoperative complication rates and the recurrence rates it was 12.6% (range: 0–61%). Some studies revealed extremely good (postoperative complication rate 7%, recurrence rate 10%) (24), others extremely poor (postoperative complication rate 72%, recurrence rate 61%), outcomes (25).The authors of the publication with the best outcomes attributed the good results to the use of a wide overlap, avoidance of dissection in the abdominal wall and coverage of the mesh with the peritoneal hernia sac or defect closure (24). Hence, no clear picture can be identified. Further studies are urgently needed to better ascertain the role of open IPOM with bridging. However, comparison of the literature data on the outcomes of the open IPOM vs. sublay technique did not reveal any essential difference (sublay: mean postoperative complication rate 18.6% (range 8.0–26%), mean recurrence rate 13.5% (range 1.6–32%)] (29). Only on evaluation of upward-deviating maximum values is a trend toward better outcomes for the sublay technique demonstrated (29).

**Table 2:** Open intraperitoneal onlay mesh with bridging for incisional hernia repair.

**Authors**	**Study design**	**Patients**	**Hernia type**	**Inclusion/Exclusion**	**Mesh type**	**Postoperative complications**	**Hospital stay**	**Recurrence**
**A**								
(19)	Retrospective observational study	*n* = 30	Incisional	Including lateral and recurrent incisional hernias	Polypropylene + ePTFE	Infection rate 3.3%	–	6.6% (*n* = 2) after 23 and 24 months
(20)	Retrospective observational study	*n* = 128	Incisional	60.2% recurrent hernias	Mersilene *n* = 95 ePTFE *n* = 33	25.6%	Mean 11.5 days (range: 6–60 days)	16% in a mean follow-up time of 48 months (range: 12–96 months)
(21)	Retrospective observational study	*n* = 53	Incisional	28% recurrences	ePTFE	Wound infection 3.7%	Average hospital stay 9 days (range: 4–20 days)	7.5%in follow-up of 7–36 months
(22)	Retrospective observational study	*n* = 186	Incisional	2.2% emergency cases. Primary incision median 85.5%, transverse 14.5%	Polyester 87%, Polypropylene 10.4%, Composite 2.6%	Early and late wound infections 11.7%, late occlusions 6.3%	–	16% in a mean follow-up time off 77 months
(23)	Prospective observational study	*n* = 61	Incisional	Defect size ≥ 10 cm, multiorificial defects, recurrence after retromuscular mesh included	Polypropylene + ePTFE, Composix	21%	Median: 13 days (range 6–70 days)	5% in a mean follow-up time of 35 months (range 8–88 months)
(25)	RCT	*n* = 18	Incisional	Clean or clean-contaminated conditions, no corticosteroids included	ePTFE, Dual mesh plus	72%	–	61% in a mean follow-up time of 22 months (range 6–36 months)
(24)	Prospective observational study	*n* = 455	Incisional	69% recurrent hernias	Polypropylene + ePTFE, Composic	7%	Average length of hospital stay 1.1 days	1% at a mean follow-up of 29.3 months
**B**								
(26)	Retrospective observational study	*n* = 100	Incisional	51% recurrent hernias	Parietex Composite, Polyester with absorbable hydrophilic film made of collagen, polyethyleneglycol and glycerol	12%	Mean: 7 days (range 5–17 days)	6% after 6 months follow-up
(27)	Retrospective observational study	*n* = 72	Incisional	Recurrent hernias 50% included	Dual mesh ePTFE	13.9% (7 seromas, 2 deep wound infections, 1 mesh removal)	Median: 6.4 ± 2.5 days (range 4–20 days)	4.1% in a follow-up time ranging 5–45 months
(28)	Retrospective observational study	*n* = 36	Incisional	Recurrent hernia included	Proceed, Polypropylene, and oxidized regenerated cellulose and polydioxanone	30.6%	-	0% in a median follow-up time of 28 months (range 6–36 months)

### Open intraperitoneal onlay mesh with defect closure by a myoaponeurotic flap in incisional hernia repair

To avoid a bridging situation in the open IPOM operation, techniques were introduced for defect closure in open IPOM (30). Arnaud et al. (30) first reported on an open IPOM technique with defect closure by means of a myoaponeurotic flap according to Welti and Eudel: “After excision of the scar, the herniated sac is exposed and the adjacent anterior fascia is cleared of subcutaneous tissue up to 10–15 cm from the ring of the hernia sac. The sac is then excised and intestinal adhesions dissected free to facilitate the placement of the mesh at least 10 cm from the edge of the hernia neck. The mesh is secured to the musculofascial wall by through-and-through-non absorbable sutures” (30). “The anterior lamina of the rectus sheath is incised longitudinally 4 cm back from its medial edge bilaterally. Both aponeurotic flaps are then reflected inward and sutured by interrupted absorbable stitches” (30). The publications addressing this technique report much lower postoperative complication rates and recurrence rates (30, 31, 32) (Table 3). It appears that defect closure results in both fewer postoperative complications and recurrences. This concords with the existing data showing that defect closure in incisional hernia repair assures better outcomes (13).

**Table 3:** Open intraperitoneal onlay mesh with defect closure by a myoaponeurotic flap for incisional hernia repair.

**Authors**	**Study design**	**Patients**	**Hernia type**	**Inclusion/Exclusion**	**Mesh type**	**Postoperative complications**	**Hospital stay**	**Recurrence**
(30)	Prospective observational study	*n* = 250	Incisional	48%n re-recurrences included	Dacron	Mortality 0.8%, superficial wound infection 2%, deep infection 2%, mesh removal 1.2%	–	3.2% in a mean follow-up time of 8.1 years (range 2–14 years)
(31)	Prospective observational study	*n* = 350	Incisional	20 patients had additional surgical procedures	Dacron	Mortality 0.6%, wound complications 4.0%	–	3.1% in a mean follow-up time of 8.1 years (range 2–14 years)
(32)	Prospective observational study	*n* = 280	Incisional	–	Parietex composite	Mortality 0.35%, subcutaneous surgical site infection 2%, deep infection 2%, mesh removal 1%	–	3.2%

### Open intraperitoneal onlay mesh with defect closure in modified sublay technique for incisional hernia repair

Another variant of the open IPOM technique aimed at defect closure involves a modification with the sublay technique (33). “Skin and subcutaneous flaps are then developed on both sides to allow for medialization of the rectus muscles and fascia over the mesh” (33). “The mesh is placed within the abdomen and secured with u-sutures” (33). The fascia and excess hernia is then closed over the mesh” (33). “This also allows for return of the rectus muscles to the midline thus restoring the normal architecture of the abdominal wall” (33). The outcomes with this technique (Table 4) are also highly variable and no better than those achieved with the modification involving the myoaponeurotic flap (33, 34).

**Table 4:** Open intraperitoneal onlay mesh with defect closure by sublay-technique for incisional hernia repair.

**Authors**	**Study design**	**Patients**	**Hernia type**	**Inclusion/Exclusion**	**Mesh type**	**Postoperative complications**	**Hospital stay**	**Recurrence**
(34)	Retrospective observational study	*n* = 81	Incisional	54% had undergone prior ventral/incisional hernia repair	PTFE in 83%, Prolene in 12%	Abdominal wall abscess 12%, cellulitis without abscess 4%, hematoma 20%	Average length of stay 5.8 ± 12 days	15% in a mean follow-up time of 30 ± 24 months
(33)	Retrospective observational study	*n* = 115	Incisional	34 recurrent hernias included	ePTFE, coated polyester, coated polypropylene, biologic mesh	Overall 26%, Seroma 16%	–	3.4% in a mean follow-up time of 363 days (range 15–2.212 days)

### Open intraperitoneal onlay biological mesh with defect closure by a component separation technique in contaminated ventral and incisional hernias

In a prospective multicenter study on repair of contaminated ventral hernias, 26 patients were treated by means of a component separation technique and intraperitoneal placement of a biological mesh (Strattice) (35). All patients had a defect closure and reinforcement of the repair with an appropriately sized piece of a biologic mesh with at least 3–5 cm of fascial overlap (35). The rate of wound infection was 30%, wound dehiscence 15%, seroma 15% and hematoma 8% (35). The recurrence rate after 1 year was 30%. No significant differences were noted vs. positioning of the biological mesh in the retrorectus layer (35).

### Outcome data for open intraperitoneal onlay mesh in registries

Of the 1,495 open incisional hernia repairs registered in the Danish Hernia Database, 258 (22.9%) were performed in the open IPOM technique (36). During the same period of time 1,763 incisional hernia laparoscopic repairs were carried out. Multivariate analysis revealed that younger age, open repair, hernia defects >7 cm, and onlay or intraperitoneal mesh positioning in open repair were significant risk factors for poor late outcomes (*p* < 0.05) (36). Sublay mesh position reduced the risk of reoperation for recurrence after open repairs (36).

### Assessment of the open intraperitoneal onlay mesh technique in systematic reviews and meta-analyses

In a Cochrane database systematic review comparison of onlay vs. intraperitoneal mesh position in open incisional hernia repair revealed that there were non-significantly fewer hernia recurrences, less seroma formation and more postoperative pain in the intraperitoneal group (37).

The findings of three further systematic reviews and meta-analyses must be analyzed in a critical light with respect to the key question addressed here since they included studies with primary abdominal wall hernias and incisional hernias, i.e., mixed patient collectives, as well as the laparoscopic technique (38–40). Numerous studies have demonstrated that the outcomes for repair of primary abdominal wall hernias and incisional hernias differ highly significantly and therefore should not be combined (41–45). Moreover, the mesh position as underlay was not defined exactly. These may also have included preperitoneal mesh placements (16). Studies with biological meshes were also included (38).

### Mean values of postoperative complications and recurrence rate

Under consideration of all analyzed studies the mean value for the postoperative complications is 20.4% with a range of 3.3 and 72% and for the recurrence rate 12.6% with a range of 0–61%.

## Discussion

While in an Expert Consensus Guided by Systematic Review preference is given to the sublay mesh position for repair of incisional hernia, the open intraperitoneal onlay mesh (IPOM) technique is, nonetheless, deemed useful in certain clinical situations (13). Therefore, this present review of the literature collates and analyzes the data available on the open IPOM technique. Comparison of the open with the laparoscopic technique reveals significant advantages for the laparoscopic procedure. Since in the guidelines the use of the laparoscopic IPOM technique is recommended only for a defect size of up to 10 cm (8–12), laparoscopic repair should be given preference for defects up to that size. Accordingly, the open IPOM technique in addition to other procedures tends to be used for large incisional hernias (14, 15). In general defect closure of large incisional hernias is not possible, thus creating a bridging situation as reported on in the majority of studies on the open IPOM. The outcomes demonstrate high variance. For example, for open IPOM with bridging situation postoperative complication rates of between 3.3 and 72.0% and mean value of 20.4% are identified, and for the recurrence rate of between 0 and 61.0% with mean value of 12.6%.The outcomes are very diverging. These inconsistent outcomes are probably explained by the fact that the open IPOM technique represents a very heterogeneous group. In some cases, the results derive from centers and surgeons dedicated to refining the technique, using it as the first-hand choice with great volume and awareness of the anatomical circumstances [e.g., (19–24, 26, 28, 30–32)]. In other cases, the open IPOM is applied as a desperate solution to solve a complex problem when there is no other alternative due to anatomical conditions after previous surgery [presumably references (2, 4, 17, 18, 36, 39)].

Comparison of these findings with the outcomes reported in the literature for sublay repair of incisional hernia reveals that in the case of the sublay technique the postoperative complication rates are between 8.0 and 26.0%, with a mean value of 18.6%, and recurrence rates of between 1.6 and 32.0%, with a mean value of 13.5% (29). Hence, only in respect of the extreme values are the outcomes better for the sublay technique. Likewise, registry data demonstrate somewhat more favorable outcomes for the sublay technique.

Therefore, further comparative studies are urgently needed to ascertain the role of the open IPOM technique in incisional hernia repair. That is borne out in particular in the study by Iannitti et al. (24) with a large sample size (*n* = 455) and a low postoperative complication rate of 7% and recurrence rate of 1% at a mean follow-up of 29.3 months. In the technique described by Iannitti et al. (24) dissection in the abdominal wall was avoided and attention paid to the provision of an appropriately large mesh overlap. Furthermore, the mesh was covered with at least the peritoneal hernia sac (24).

Attention should be paid to these technical aspects when implementing the open IPOM technique.

Modifications of the open IPOM technique are aimed at mesh-based defect closure through a combination with a myoaponeurotic flap or closure of the anterior lamina of the rectus sheath as used in the sublay technique. However, both techniques require dissection in the abdominal wall, albeit to a lesser degree when using myoaponeurotic flaps. That also no doubt explains the more favorable outcomes of open IPOM repair by means of a myoaponeurotic flap compared with closure of the anterior lamina of the rectus sheath. Likewise, comparison of outcomes of open IPOM technique with bridging reveals lower postoperative complication and recurrence rates for the modification with myoaponeurotic flaps. However, to date that technique has only been used and extensively reported by a French working group.

From the available meta-analyses and systematic reviews it is almost impossible to generate further data on open IPOM outcomes since they include joint evaluation of primary abdominal wall hernias and incisional hernias, open and laparoscopic techniques as well as synthetic and biological meshes. Hence, interpretation of outcomes is very difficult.

In summary, it can be stated that the open IPOM is clearly inferior to the laparoscopic technique but achieves quite acceptable outcomes compared with the open sublay technique. It appears that outcomes can be further improved through the use of a wide overlap, avoidance of dissection in the abdominal wall and defect closure. Further studies using a standardized open IPOM technique are urgently needed.

## Author contributions

FK: literature search, literature analyses, publication concept, and publication draft; BL: literature search, literature analyses, publication concept, and critical review of the publication draft.

### Conflict of interest statement

The authors declare that the research was conducted in the absence of any commercial or financial relationships that could be construed as a potential conflict of interest.
